# Maternal Eating Styles and Restrictive Feeding Practices: Indirect Effects Through Perceived Child Appetite and Weight Concern

**DOI:** 10.3390/nu17243933

**Published:** 2025-12-16

**Authors:** Carla Ugarte Pérez, Claudia Cruzat Mandich, Camila Oda-Montecinos, Fernanda Díaz Castrillón, Álvaro Quiñones Bergeret, Antonio Cepeda-Benito

**Affiliations:** 1 Centro de Estudios de la Conducta Alimentaria, Escuela de Psicología, Universidad Adolfo Ibáñez, Avenida Diagonal Las Torres 2640, Peñalolén 7941169, Santiago, Chile; claudia.cruzat@uai.cl (C.C.M.); camilamarta.oda@unir.net (C.O.-M.); fernanda.diaz@uai.cl (F.D.C.); 2 Facultad de Ciencias de la Salud, Universidad Internacional de la Rioja, Avenida de la Paz 137, 26006 Logroño, La Rioja, Spain; 3 Departamento de Ciencias Sociales, Universidad de Tarapacá, Sede Iquique, Av. La Tirana #4802, Iquique 1101783, Región de Tarapacá, Chile; aquinones@academicos.uta.cl; 4 Department of Psychological Science, University of Vermont, 2 Colchester Avenue, Burlington, VT 05405, USA

**Keywords:** maternal eating styles, parental feeding practices, restrictive feeding, child eating behavior, obesity risk, preschool children

## Abstract

**Background:** Parents play a central role in shaping children’s eating behaviors. While previous research has documented associations between parental attitudes and feeding practices, fewer studies have examined how mothers’ own eating styles may contribute to their perceptions of their children’s eating attitudes and behaviors and how these may influence subsequent feeding practices. **Objectives:** To our knowledge, this is the first study to examine whether mothers’ eating styles predicted their self-reported restrictive feeding practices indirectly through their perceptions of their children’s appetite and subsequently through their concern about their children’s weight. **Methods:** A total of 488 mothers (M_age = 33.87 years, SD = 4.81, range = 20–49) of children aged 2–7 years (M_age = 3.85 years, SD = 1.33) completed self-report measures, including the Dutch Eating Behavior Questionnaire (DEBQ) for maternal eating styles, the Child Feeding Questionnaire (CFQ) for parental concerns and restrictive practices, and the Children’s Eating Behavior Questionnaire (CEBQ) for perceptions of child eating attitudes. Structural equation modeling (SEM) was employed to test the hypothesized mediation model, with model fit evaluated using CFI, TLI, RMSEA, and SRMR indices. **Results:** Our proposed model demonstrated good fit (CFI = 0.94, RMSEA = 0.07) and showed that maternal eating styles were positively associated with perceived child appetite (β = 0.44, *p* < 0.001). Perceived appetite predicted both maternal concern about child weight (β = 0.39, *p* < 0.001) and restrictive feeding practices (β = 0.28, *p* < 0.001), while maternal concern strongly predicted restriction (β = 0.65, *p* < 0.001). The total indirect effect from maternal eating styles to restriction was significant (β = 0.23, *p* < 0.001), and the model explained 56% of the variance in restrictive feeding. **Conclusions:** Our findings suggest that maternal eating styles may bias mothers’ perceptions of their children’s appetite and indirectly influence restrictive feeding practices primarily through increased concern about child weight. Given the cross-sectional design, reliance on maternal self-report, and online convenience sampling, results should be interpreted cautiously. Nonetheless, the study provides the first evidence for a sequential pathway linking maternal eating styles, child appetite perceptions, and weight concern to restrictive feeding, highlighting cognitive and perceptual processes as intervention targets.

## 1. Introduction

Childhood obesity and unhealthy eating behaviors remain pressing public health concerns globally and in Chile. According to the World Health Organization (WHO), more than 39 million children under the age of five were overweight or obese in 2020, with prevalence rates steadily increasing across both developed and developing nations [1]. Chile, in particular, ranks among the countries most affected in South America [2]. According to national monitoring data from the Junta Nacional de Auxilio Escolar y Becas (JUNAEB) [3], approximately half of Chilean children entering kindergarten are classified as overweight or obese. These alarming statistics highlight the urgent need to identify psychosocial factors contributing to early eating behaviors and weight-related outcomes.

Parental influence is among the strongest determinants of children’s dietary habits, food preferences, and weight outcomes [4]. The home food environment and parental feeding practices shape not only what children eat but also how they develop attitudes toward food and intake self-regulation [5]. Feeding strategies such as restriction, pressure to eat, and monitoring have been consistently linked with children’s eating patterns and risk of obesity [6,7]. Importantly, beyond specific feeding practices, parental attitudes and dispositions toward child feeding play a crucial role. In particular, parents who are highly concerned about their child’s weight may engage in restrictive feeding to prevent weight gain, yet such strategies can paradoxically increase children’s preference for restricted foods and reduce their ability to regulate appetite [8,9].

A growing body of research has emphasized that parents’ own eating behaviors and tendencies are integral to understanding how they approach child feeding [10,11]. Maternal eating styles—specifically restrained, emotional, and external eating—have been conceptualized as dispositional tendencies that influence how mothers perceive and respond to food-related cues [12,13]. Mothers high in restrained eating may be particularly vigilant about their children’s food intake, while those high in emotional or external eating may misinterpret children’s hunger or satiety cues [14,15]. Such tendencies shape maternal subsequent feeding practices [16,17].

Another key factor in this dynamic is how mothers perceive their children’s eating attitudes and appetitive traits. The Children’s Eating Behavior Questionnaire (CEBQ) [18] captures parental perceptions of traits such as food responsiveness, enjoyment of food, and satiety responsiveness. These perceptions may or may not reflect children’s actual eating behaviors but stands to reason they likely guide how parents monitor and regulate their children’s feeding. For instance, perceiving a child as highly food responsive (i.e., having a strong drive to eat) may prompt greater use of restrictive feeding practices [17]. Although previous research has examined unique pathways linking parental and child eating-related variables, no study to our knowledge has proposed a comprehensive model that attempts to explain how parental eating styles influence restrictive feeding practices through potential mediators such as children’s characteristics and the concerns such characteristics may arise in the parents. For example, Rodgers et al. [16] found that specific maternal feeding practices—particularly instrumental and emotional feeding—correlated with subsequent increases in children’s obesogenic eating behaviors. These authors also found that maternal monitoring was correlated with decreases in food-approach behaviors. However, these authors did not consider how the mothers’ own dysregulated eating might have shaped either or both their perceptions of their children’s obesogenic eating and their restrictive feeding and monitoring. In contrast, Pickard et al. [19] demonstrated that parents’ own eating profiles were directly linked to their children’s latent eating profiles, with these associations mediated by feeding practices such as using food for emotional regulation and maintaining a healthy food environment. However, this study did not incorporate parents’ perceptions of children’s appetitive traits or weight-related concerns—nor provided a compelling argument for hypothesizing why feeding practices influenced parental perceptions of their children’s appetitive drives rather than the reverse. Complementary evidence from Haycraft and Blissett [15] showed that higher parental BMI was associated with more controlling feeding, suggesting that parents’ own weight and eating tendencies may influence mealtime control. Finally, Russell et al. [17] provided evidence that parents adjust controlling feeding practices in response to child characteristics but did not explore what cognitive and motivational processes may precede or mediate the relationship between the child’s characteristics and the parental behavior.

Taken together, these studies show relationships between parental eating profiles and/or indicators of their appetitive drives [15,16,19], children’s appetitive drive and obesogenic traits (e.g., [16,17,19]), and parental feeding practices (e.g., [15,16,17,18,19]). Except for Russell et al. [17], all conceptualize parental variables and behaviors as antecedents of children’s measured traits and behaviors. In addition, these relationships have generally been examined at the bivariate rather than mediational levels (although there are exceptions such as Pickard et al. [19]). We are fairly confident that an integrated, sequential model connecting parental eating styles, children’s appetitive drive, parental concern, and parental restrictive feeding remains untested. Addressing this gap is particularly relevant early on, when children are highly dependent on caregivers for food provision and when foundational self-regulation patterns in eating begin to be established (preschool and kindergarten years). Thus, building on the above cited literature, the present study addresses a critical gap by modeling a sequential, cognitively mediated pathway in which maternal eating styles shape mothers’ perceptions of their children’s appetite, which subsequently fuel concerns about weight and then their restrictive feeding responses.

### Study Objectives and Hypotheses

The present study aimed to test a structural equation model examining whether maternal eating styles predicted restrictive feeding practices indirectly through mothers’ perceptions of their children’s appetite and their subsequent concern about child weight. Based on previous findings, we hypothesized that:

1. Higher levels of dysregulated maternal eating (DEBQ-derived latent factor) would predict higher perceived child food drive (CEBQ-derived latent factor).
2. Greater perceived child food drive would predict higher maternal worry about child weight (CFQ-derived latent factor).
3. Both perceived child food drive and maternal worry would, in turn, predict more maternal control through restricting, prohibiting and monitoring feeding (CFQ-derived latent factor).
4. The relationship between dysregulated maternal eating and maternal control would be primarily indirect, operating sequentially through perceived child food drive and maternal worry.

## 2. Materials and Methods

### 2.1. Participants

Participants were 488 mothers of children aged 2 to 7 years. Mothers’ mean age was 33.87 years (SD = 4.81; range = 20–49), and their mean body mass index (BMI) was 26.95 kg/m^2^ (SD = 5.52; range = 15.92–52.07), placing the sample, on average, in the overweight category according to World Health Organization (WHO) criteria. On average, mothers reported a median of 2 children (range = 1–12). Children’s mean age was 3.85 years (SD = 1.33).

With respect to nationality, the majority of participants identified as Chilean (93.6%), with additional, similarly distributed representation from Argentina, Venezuela, Colombia, Peru, and Bolivia. Regarding maternal education, 3.4% had basic education, 24.0% secondary, 49.4% higher education, and 23.2% postgraduate training. In terms of occupational status, 40.4% were salaried employees, 22.7% were self-employed, and 18.9% performed unpaid work. Additionally, 11.3% were unemployed, 3.9% were not engaged in labor activity, and 2.9% were students.

Participants lived in 109 comunas across all 16 regions of Chile, from the far north to the far south. Most resided in the Metropolitan Region (24.7%) and Valparaíso (3.9%), with the remainder distributed across the northern, central and southern regions.

### 2.2. Procedure

Participants were recruited online via targeted advertisements and posts on social media platforms (e.g., Facebook, Instagram) directed to Chilean parenting communities. Eligibility criteria required participants to be mothers of at least one child aged 2–7 years and to be primarily responsible for feeding that child.

After clicking on the study link, interested participants were presented with an electronic informed consent form describing the purpose of the study, its voluntary nature, and data protection procedures. Only those who provided consent were permitted to proceed to the survey. Data were collected via a self-administered questionnaire hosted on a secure online platform and stored in password-protected files accessible only to authorized members of the research team.

No exclusion criteria beyond the predefined child age range were applied. All participants who met the eligibility criteria and provided valid data were included in the analyses. Thus, no post hoc participant exclusions were performed.

To encourage participation, respondents were entered into a raffle for one of 30 electronic gift cards valued at 45,000 Chilean pesos (CLP), about 45 US dollars. Gift cards were delivered via an email address provided by the participants who wanted to enter the raffle. No other contact information was collected, and the emails were kept separate from the analyzed data.

### 2.3. Ethics

The study was conducted in accordance with the Declaration of Helsinki and was approved by the Ethics Committee of the School of Psychology, Universidad Adolfo Ibáñez. All participants provided informed consent prior to participation.

### 2.4. Measures

Maternal Eating Styles. The Dutch Eating Behavior Questionnaire (DEBQ) [12] was used to assess three maternal eating style dimensions: *Restrained Eating* (10 items; e.g., “Do you deliberately eat less in order not to become heavier?”; α = 0.86), *Emotional Eating* (13 items; e.g., “Do you have a desire to eat when you are irritated?”; α = 0.93), and *External Eating* (10 items; e.g., “If food smells and looks good, do you eat more than usual?”; α = 0.84). Items are rated on a 5-point Likert scale ranging from 1 (“never”) to 5 (“very often”), with subscale scores computed as the mean of their respective items. The DEBQ has demonstrated strong internal consistency and factorial validity in Chilean and broader Latin American samples.

In line with the aims of the present study, the three DEBQ subscales were modeled as indicators of a single latent construct representing Dysregulated Maternal Eating. Although the DEBQ dimensions are conceptually distinct, they each reflect patterns of eating that increase vulnerability to overeating, loss of control, and weight gain. Treating them as indicators of a singular latent construct provides a parsimonious way to capture an overall tendency toward dysregulated eating. This modeling decision was further supported empirically, as all three subscales loaded significantly on the latent factor, with standardized loadings ranging from modest to strong, indicating meaningful shared variance across indicators.

Child Feeding Attitudes and Practices. The Child Feeding Questionnaire (CFQ) [6,7] was employed to assess mothers’ perceptions of child weight, concerns about child weight, and feeding-related practices. Four subscales were used: *Concern about Child Weight* (3 items; e.g., “How concerned are you about your child eating too much when you are not around?”; α = 0.79), *Restriction* (8 items; e.g., “I intentionally keep some foods out of my child’s reach”; α = 0.82), *Monitoring* 3 items; e.g., “How much do you keep track of the high-fat foods your child eats?”; α = 0.75); and *Prohibition* (3 items; e.g., “I forbid my child from eating certain foods”; α = 0.77).

In the SEM, *Maternal Worry* was defined by the *Concern* items, and *Maternal Control* was defined by the *Restriction*, *Monitoring*, and *Prohibition* subscales of the CFQ. Although these practices are behaviorally distinct, they share a common regulatory function—namely, control food intake through the intentional restriction, limitation, or supervision of children’s food intake. Modeling them as indicators of a single regulatory construct therefore provides a theoretically coherent and parsimonious representation of maternal control over feeding behavior. This modeling decision was also supported empirically, as all three indicators loaded significantly on the latent factor, indicating meaningful shared variance.

Perceived Child Eating Attitudes. The Children’s Eating Behavior Questionnaire (CEBQ) [18] assesses parental perceptions of children’s appetitive traits across eight subscales. The present study focused on the food pro-ingestive dimension, which integrates the subscales *Food Responsiveness* (5 items; e.g., “My child is always asking for food”; α = 0.84), *Emotional Overeating* (4 items; e.g., “My child eats more when worried”; α = 0.76), and *Enjoyment of Food* (4 items; e.g., “My child loves food”; α = 0.88). Responses were given on a 5-point Likert scale (1 = “never” to 5 = “always”). These subscales jointly defined the latent variable *Child Food Drive*, representing maternal perceptions of children’s appetite and motivation to eat. All measures were validated Spanish-language versions with strong psychometric properties and demonstrated reliability in Chilean and Latin American populations.

### 2.5. Data Analysis

Structural equation modeling (SEM) analyses were conducted in *R* using the *lavaan* package [20]. Models were estimated using maximum likelihood with robust standard errors (MLR), which provides Huber–White robust SEs and a Satorra–Bentler (SB)-scaled χ^2^ test of model fit [21]. Model adequacy was evaluated using robust comparative fit indices (CFI, TLI), the robust RMSEA with 90% confidence intervals, and the SRMR. AIC and BIC were examined for model parsimony.

Four latent variables were specified:

- Maternal Eating Styles: DEBQ Restrained, Emotional, and External eating total factor scores.
- Child Drive: CEBQ Food Responsiveness, Enjoyment of Food, and Emotional Overeating total factor scores
- Maternal Worry: The three CFQ concern-factor items.
- Maternal Control: CFQ Restriction, Monitoring, and Prohibition factor-total scores.

**Figure 1:**
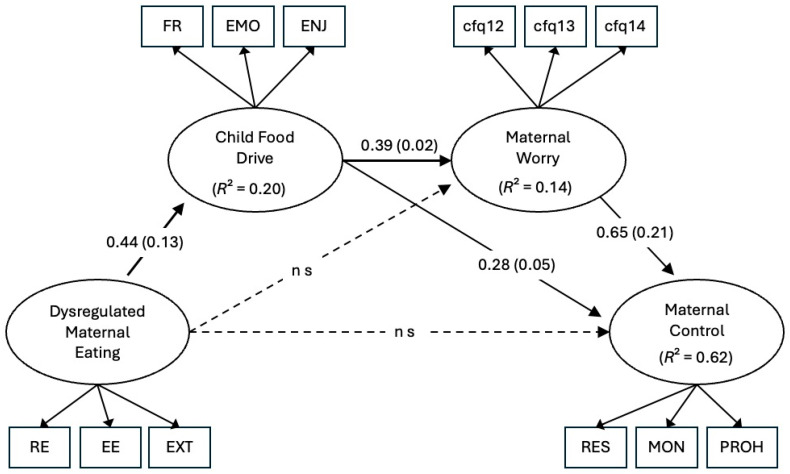
Reduced structural equation model showing standardized path coefficients (β) with standard errors in parentheses and R^2^ values for each endogenous latent variable. Solid arrows represent significant paths (*p* < 0.001); dashed arrows indicate nonsignificant paths omitted from the reduced model. Dysregulated Maternal Eating was indicated by DEBQ Restrained (RE), Emotional (EE), and External (EXT) eating. Child Food Drive was indicated by CEBQ Food Responsiveness (FR), Enjoyment of Food (ENJ), and Emotional Overeating (EMO). Maternal Worry was measured with CFQ concern items (cfq12–14), and Maternal Control was indicated by CFQ Restriction (RES), Monitoring (MON), and Prohibition (PROH). Prior to model estimation, distributions of all observed indicators were inspected for skewness, which ranged from 0.04 to 1.23, and kurtosis, which ranged from −1.21 to 0.79, indicating no severe departures from normality and fell within acceptable ranges for SEM with robust maximum likelihood estimation (see Supplemental Table S1). Missing data were minimal (<1% across variables) and were handled using mean substitution.

Two nested models were tested: The full model included all direct and indirect paths from Dysregulated Maternal Eating to Maternal Control sequentially through Child Food Drive and Maternal Worry (Figure 1). The reduced model retained the identical measurement structure but omitted two nonsignificant direct paths (Dysregulated Maternal Eating → Maternal Worry; Dysregulated Maternal Eating → Maternal Control). Indirect and total effects were computed within *lavaan* and tested using the delta method under MLR. Model comparison relied on the SB-scaled χ^2^ difference test (lavTestLRT) and inspection of robust fit indices and AIC/BIC values.

To evaluate the robustness of the parameter estimates to distributional assumptions, the model was estimated using both maximum likelihood (ML) and robust maximum likelihood (MLR) estimators. The pattern and magnitude of the parameter estimates were virtually identical across estimators, indicating that the results were not materially affected by deviations from multivariate normality.

As an additional robustness check, we examined whether the measurement model demonstrated invariance across boys and girls. We tested configural, metric, and scalar invariance by estimating multigroup CFA models and comparing robust fit indices and SB-scaled χ^2^ difference tests. Invariance was evaluated using conventional ΔCFI ≤ 0.01 and ΔRMSEA ≤ 0.015 thresholds.

We also conducted sensitivity analyses by testing two alternative recursive orderings. Model B placed Maternal Worry before Child Food Drive, and Model C conceptualized Maternal Control as the most immediate predictor of Child Food Drive. Each alternative model was estimated in full form and in reduced form with nonsignificant paths removed. Additionally, two alternative parallel models were tested. Model D examined the indirect effect of Dysregulated Maternal Eating on Maternal Control through Child Food Drive and Maternal Worry operating in parallel, whereas Model E tested the indirect effect of Dysregulated Maternal Eating on Child Food Drive through Maternal Control and Maternal Worry.

## 3. Results

### 3.1. Model Fit

The reduced model provided a good to excellent fit to the data, χ^2^(50) = 163.60, *p* < 0.001, CFI = 0.94, TLI = 0.92, RMSEA = 0.07, SRMR = 0.06 [22]. Model comparison indicated that removing the two nonsignificant direct paths from Dysregulated Maternal Eating to Maternal Worry and to Maternal Control did not degrade fit, Δχ^2^(2) = 1.07, *p* = 0.59, and moderately improved AIC (29,738 vs. 29,735) and BIC (29,914 vs. 29,903). See Table 1 for complete fit indices.

### 3.2. Structural Paths

Standardized path coefficients for the reduced model are shown in Table 2. Dysregulated Maternal Eating significantly predicted greater Child Food Drive (β = 0.44, *p* < 0.001). In turn, Child Food Drive predicted both higher Maternal Worry (β = 0.39, *p* < 0.001) and higher Maternal Control (β = 0.28, *p* < 0.001). Maternal Worry also strongly predicted Maternal Control (β = 0.65, *p* < 0.001).

Indirect effects indicated two significant mediated pathways linking Dysregulated Maternal Eating to Maternal Control: (a) through Child Food Drive (β = 0.12, *p* < 0.001) and (b) sequentially through Child Food Drive → Maternal Worry (β = 0.11, *p* < 0.001). The combined indirect effect was significant (β = 0.23, *p* < 0.001). The total effect of maternal eating styles on restriction remained significant (β = 0.17, *p* = 0.008). The reduced model accounted for substantial proportions of variance in all endogenous constructs: R^2^ = 0.203 for Child Food Drive, R^2^ = 0.142 for Maternal Worry, and R^2^ = 0.616 for Maternal Control. Indicator factor loadings and residual variances are reported in Supplemental Table S2.

### 3.3. Measurement Invariance Across Sex

Measurement invariance testing supported configural, metric, and scalar invariance across boys and girls. Robust CFI, TLI, and RMSEA values were highly similar across increasingly constrained models, and ΔCFI and ΔRMSEA values remained well within recommended thresholds. SB-scaled χ^2^ difference tests were nonsignificant for both configural vs. metric (*p* = 0.157) and metric vs. scalar (*p* = 0.595) comparisons. These findings indicate that the latent constructs were measured equivalently across sex groups, and the structural paths can be interpreted without concern for measurement bias. Full invariance results are presented in Supplemental Table S4.

### 3.4. Alternative Model Tests

All alternative recursive orderings yielded global fit indices nearly identical to those of the proposed model (e.g., |ΔCFI| < 0.001; AIC/BIC differences near or <2), a pattern consistent with alternative recursive specifications often reproducing similar covariance structures in cross-sectional SEM (Supplemental Table S3). The parallel models produced comparatively poorer fit, particularly Model E, which placed Child Food Drive as the outcome. Given that our hypothesized ordering was theoretically and empirically grounded and no alternative model provided a meaningfully superior fit, we retained the reduced proposed model as the final analytic model.

## 4. Discussion

This study tested an integrative model linking maternal eating styles to restrictive feeding practices through mothers’ perceptions of their children’s appetitive traits and concern about child weight in a Chilean sample. Structural equation modeling revealed that while the direct effect was nonsignificant, maternal dysregulated eating (restrained, emotional, and external eating) was indirectly associated with maternal control of feeding (restriction, prohibition, and monitoring) via perceived child food drive (food responsiveness, emotional eating, and enjoyment of food) and maternal worry over their child gaining weight. These findings highlight the role of maternal perceptions and cognitions as mechanisms through which parental traits shape feeding control.

### 4.1. Interpretation and Theoretical Implications

Consistent with our hypotheses, mothers who reported greater restrained, emotional, or external eating perceived their children as having stronger pro-ingestive drives (e.g., food responsiveness, enjoyment of food, and emotional overeating). This association supports the idea that parents’ own eating tendencies color their perceptions of their children’s appetite cues, a pattern previously observed by Temmen et al. [23]. Both studies suggest that maternal eating tendencies, whether emotional or externally driven, influence how parents interpret and respond to their children’s hunger and satiety signals—often amplifying perceived risk of overeating. These biases likely arise through modeling or projection, whereby parents’ own preoccupations with food and weight heighten their sensitivity to their children’s interest in food [15].

Perceived child drive, in turn, predicted both heightened maternal concern about their child weight and greater restrictive, limiting, and monitored feeding, consistent with prior evidence that parental perceptions of high appetite or weight risk trigger controlling feeding practices [8]. The strong association between maternal concern and restriction underscores the proximal role of worry about child weight gain in eliciting restrictive control. Collectively, these patterns align with cognitive-behavioral models proposing that parental cognitions—such as perceived appetite risk and weight concern—mediate the link between parental eating tendencies and feeding practices [18]. The absence of a direct path from dysregulated maternal eating to maternal control further suggests that interventions should target perceptual and cognitive processes rather than focusing exclusively on behavioral regulation of feeding.

### 4.2. Relation to Previous Research

Previous studies have examined individual components of this pathway but rarely within a single integrated framework. For example, parents’ perceptions of their children’s weight, rather than actual BMI, has been found to predict restrictive and pressuring practices [9]. Similarly, parents’ concern about weight and diet quality appears to predict their use of controlling strategies, particularly among overweight children [8]. Studies of parental eating patterns also support indirect influences: Temmen et al. [23] showed that maternal emotional and external eating predicted greater “feeding to soothe” during infancy, which in turn increased infant food responsiveness. Likewise, Singh et al. [24] reported that maternal own restrained eating was associated with more pressure feeding, while emotional and external eating interacted with child weight to predict contradictory concerns about under- or overeating. These findings, together with those from Pickard et al. [19], who identified parallel parent–child eating profiles mediated by feeding practices, converge to suggest that parental eating styles influence feeding behavior indirectly through perceptions and cognitions.

The present study advances this literature by modeling both perceptual (child food drive) and cognitive (weight concern) mediators simultaneously and by confirming their sequential contribution to restrictive feeding. This approach integrates prior variable-centered and person-centered findings into a single, theoretically coherent framework, extending transactional models of feeding and appetite regulation [16].

Recent work in Latin-American populations further supports the importance of these mechanisms. Mexican mothers frequently misperceive their infants’ weight and report difficulties interpreting hunger and satiety cues [25], while maternal concern about child weight has been shown to predict more coercive and controlling feeding strategies in regional samples [26]. Studies in Chile similarly demonstrate that children’s appetitive traits—particularly food responsiveness—interact with parental feeding styles to predict later weight outcomes [27]. Parallel findings from Mexico indicate modest intergenerational correspondence in appetitive traits between mothers and children [28], and prospective evidence shows that early appetite-related behaviors, such as slowness in eating, predict weight gain across infancy [29]. Together, these studies reinforce the perceptual and cognitive pathways modeled here and situate our results within a broader Latin-American context.

### 4.3. Practical and Clinical Implications

The findings have important implications for family-based interventions. Efforts to promote adaptive feeding practices should focus not only on observable behaviors but also on the parental cognitions that drive them. Helping parents recognize normal variability in children’s appetite and avoid pathologizing robust eating could reduce unnecessary restriction and meal-related tension. Psychoeducational programs might also address maternal emotional and external eating patterns that amplify weight-related worry, incorporating cognitive-behavioral or mindfulness-based strategies to recalibrate parents’ interpretations of children’s eating behavior. By targeting perceptual accuracy and emotional regulation, such interventions could disrupt a self-reinforcing loop linking maternal anxiety, child feeding control, and children’s subsequent risk of dysregulated eating.

From a practical standpoint, these results suggest that efforts to promote healthy child feeding should address not only overt feeding behaviors but also the maternal eating styles and interpretive processes that shape parents’ responses to children’s appetite cues. Preventive programs that help mothers manage emotion-driven or externally cued eating—and that reduce weight-related worry—may lessen reliance on restrictive feeding strategies that inadvertently undermine children’s capacity for self-regulation.

### 4.4. Strengths, Limitations, and Future Directions

This study contributes novel evidence from a large, non-WEIRD Latin American sample, addressing calls to diversify the populations represented in behavioral research [30,31]. Additional strengths include validated Spanish-language measures and a theoretically grounded SEM model that captures the sequential interplay of maternal eating styles, perceptions of child appetite, weight-related worry, and restrictive feeding.

Several limitations warrant consideration. The cross-sectional design prevents establishing temporal precedence, and the associations observed should be interpreted as correlational. Although all variables were assessed through maternal report, this approach is conceptually appropriate for the constructs of interest, as maternal perceptions and cognitions are the mechanisms hypothesized to influence feeding practices. Nonetheless, shared-method variance and reporting biases cannot be ruled out. Online recruitment may also introduce sampling bias by underrepresenting families with limited Internet access or those less engaged with parenting communities, and the exclusive inclusion of mothers limits generalizability to fathers or alternative caregivers. Despite these constraints, the sample displayed substantial geographic and sociodemographic heterogeneity, supporting the ecological relevance of the findings.

To further evaluate the robustness of the model, we tested several alternative recursive orderings—including those suggested by reviewers—as well as multiple parallel mediation structures. As expected in cross-sectional SEM, alternative recursive specifications produced nearly identical global fit indices. However, parallel models provided discriminating evidence: specifications placing maternal restriction prior to perceptions of child appetite showed markedly poorer fit and inadmissible estimates, reinforcing the theoretical ordering adopted in the present study. Even so, causal direction cannot be established empirically, and these findings should be interpreted with caution.

Future research should employ longitudinal and observational methods to clarify temporal ordering and bidirectionality between child appetitive traits and parental feeding behavior. Incorporating fathers and multiple caregivers integrating multi-informant and behavioral assessments and including objective indicators of children’s eating and weight regulation would strengthen inference and extend the developmental and clinical applicability of this work.

## 5. Conclusions

In summary, the findings are consistent with a model in which dysregulated maternal eating was associated with maternal restricting control over feeding indirectly through mothers’ perceptions of their children’s appetitive drive and their weight-related concern. Mothers reporting higher restrained, emotional, or external eating tended to perceive their child as more food-driven and to report greater worry about their child potential weight gain. These perceptions were in turn associated with exerting higher control and vigilance over what their child ate. These patterns highlight perceptual and cognitive processes that may be relevant targets for interventions aimed at fostering balanced, responsive feeding dynamics and supporting healthy eating development during early childhood.

## Figures and Tables

**Table 1:** Fit indices for full and reduced structural equation models.

Model	χ^2^ (df)	R-CFI	R-TLI	R-RMSEA [90% CI]	SRMR	AIC	BIC
Full	162.34 (48) ***	0.94	0.91	0.069 [0.057, 0.081]	0.056	29,738	29,914
Reduced	163.60 (50) ***	0.94	0.92	0.067 [0.055, 0.079]	0.055	29,735	29,903

Note. R-CFI = Robust Comparative Fit Index; R-TLI = Robust Tucker–Lewis Index; R-RMSEA = Robust Root Mean Square Error of Approximation; SRMR = Standardized Root Mean Square Residual; CI = confidence interval. *** *p* < 0.001. Model fit was evaluated using standard criteria: CFI and TLI ≥ 0.90 (preferably ≥ 0.95), RMSEA ≤ 0.08 (≤0.06 for close fit), and SRMR ≤ 0.08.

**Table 2:** Standardized path coefficients for the reduced structural equation model.

Path	β	SE	z	*p*
Direct effects:				
Dysregulated Maternal Eating → Child Food Drive	0.44	0.13	5.37	<0.001
Child Food Drive → Maternal Worry	0.39	0.02	6.06	<0.001
Child Food Drive → Maternal Control	0.28	0.05	4.91	<0.001
Maternal Worry → Maternal Control	0.65	0.21	12.89	<0.001
Indirect effects of Dysregulated Maternal Eating on Maternal Control through:				
Child Food Drive	0.12	0.05	3.77	<0.001
Child Food Drive → Maternal Worry	0.11	0.04	3.73	<0.001
Total indirect effect	0.23	0.08	4.16	<0.001
Total effect:				
Dysregulated Maternal Eating → Maternal Control	0.17	0.09	2.66	0.008

Note. Standardized coefficients (β) are reported. All effects are estimated from the reduced model in which nonsignificant direct paths from Dysregulated Maternal Eating to Maternal Worry and Maternal Control were omitted. Indirect effects were computed in *lavaan* using labeled parameters and tested for significance with the delta method under the MLR estimator.

## Data Availability

The original contributions presented in the study are included in the article/Supplementary Materials, further inquiries can be directed to the corresponding author.

## Supplementary Materials

The following supporting information can be downloaded at: https://www.mdpi.com/article/10.3390/nu17243933/s1, Table S1: Descriptive statistics and intercorrelations among observed variables. Table S2. (Residual Variances) and Standardized Factor-Loading Matrix of the Final Retained Sequential Model. Table S3. Model comparison statistics for proposed, alternative, and parallel models. Table S4. Measurement Invariance of the Reduced Measurement Model Across Boys and Girls.
