# Supplementary material for: Maternal Eating Styles and Restrictive Feeding Practices: Indirect Effects Through Perceived Child Appetite and Weight Concern
Source: Nutrients. 2025 Dec 16;17(24):3933. doi: 10.3390/nu17243933 (PMC12735459; doi:10.3390/nu17243933)
Supplement: Supplementary file 1 [file nutrients-17-03933-s001.zip › nutrients-4005295-supplementary.pdf]

Table S1.

Descriptive statistics and intercorrelations among observed variables.

Indicators	M	SD	*skew*	*kurt*	1	2	3	4	5	6	7	8	9	10	11	12
DEBQ_RE	25.88	8.84	0.35	-0.40	1.00											
DEBQ_EE	32.84	13.44	0.50	-0.40	0.32	1.00										
DEBQ_EXT	30.51	7.27	0.17	-0.17	0.14	0.55	1.00									
CEBQ_FR	13.82	5.23	0.69	0.15	0.12	0.33	0.34	1.00								
CEBQ_EMO	5.06	2.00	1.01	0.79	0.14	0.32	0.23	0.57	1.00							
CEBQ_ENJ	13.14	3.12	0.04	-0.16	0.02	0.09	0.10	0.51	0.31	1.00						
CFQ_12	2.86	1.38	0.18	-1.19	0.21	0.12	0.06	0.29	0.27	0.23	1.00					
CFQ_13	2.13	1.38	0.95	-0.45	0.23	0.11	0.07	0.24	0.24	0.24	0.62	1.00				
CFQ_14	2.97	1.38	0.14	-1.21	0.16	0.06	0.04	0.25	0.19	0.23	0.62	0.65	1.00			
CFQ_RES	17.74	5.21	0.04	-0.74	0.14	0.09	0.21	0.41	0.29	0.25	0.55	0.48	0.51	1.00		
CFQ_MON	12.13	3.06	-0.93	0.02	0.13	0.05	0.03	0.10	0.10	0.14	0.30	0.26	0.32	0.38	1.00	
CFQ_PROH	5.46	3.05	1.23	0.73	0.10	0.03	0.03	0.16	0.26	0.08	0.23	0.32	0.27	0.39	0.24	1.00

Notes. *M* = mean; *SD* = standard deviation; *skew* = skewness; *kurt* = kurtosis. Correlations are Pearson coefficients among observed indicators. *Dysregulated Maternal Eating*: *DEBQ* scales: DEBQ_RE (1) = Restrained Eating; DEBQ_EE (2) = Emotional Eating; DEBQ_EXT (3) = External Eating. *Child Pro-Food Drive*: *CEBQ* scales: CEBQ_FR (4) = Food Responsiveness; CEBQ_EMO (5) = Emotional Overeating; CEBQ_ENJ (6) = Enjoyment. *Maternal Worry*: *CFQ Concern about Child Weight Scale* items = CFQ_i12 (7); CFQ_i13 (8); CFQ_i14 (9). *Maternal Control*: *CFQ* scales: CFQ_RES (10) = Food Restriction; CFQ_MON (11) = Food Monitoring; CFQ_PROH (12) = Food Prohibition.

Table S2.

(Residual Variances) and Standardized Factor-Loading Matrix of the Final Retained Sequential Model.

Latent construct	Indicator	β	R^2	SE	z	p
Dysregulated Maternal Eating (1.000)	DEBQ_RE	0.345	0.119	0.484	6.31	< .001
	DEBQ_EE	0.846	0.715	0.918	12.36	< .001
	DEBQ_EXT	0.649	0.421	0.478	9.85	< .001
Child Pro-Food Drive (0.690)	CEBQ_FR	0.915	0.837	0.282	15.12	< .001
	CEBQ_EMO	0.634	0.402	0.104	10.84	< .001
	CEBQ_ENJ	0.539	0.290	0.145	10.31	< .001
Maternal Worry (0.967)	CFQ_12	0.790	0.623	0.046	21.87	< .001
	CFQ _13	0.792	0.627	0.055	18.23	< .001
	CFQ _14	0.801	0.642	0.046	21.97	< .001
Maternal Control (0.383)	CFQ_RES	0.866	0.750	0.289	9.66	< .001
	CFQ_MON	0.447	0.200	0.108	7.86	< .001
	CFQ_PROH	0.454	0.206	0.106	8.10	< .001

Note. All l β loadings are standardized from the final retained sequential SEM model with MLR.

Dysregulated Maternal Eating: DEBQ scales: DEBQ_RE = Restrained Eating; DEBQ_EE = Emotional Eating; DEBQ_EXT = External Eating. Child Pro-Food Drive: CEBQ scales: CEBQ_FR = Food Responsiveness; CEBQ_EMO = Emotional Overeating; CEBQ_ENJ = Enjoyment. Maternal Worry: CFQ Concern about Child Weight Scale items = CFQ_12, CFQ_13, CFQ_14. Maternal Control: CFQ scales: CFQ_RES = Food Restriction; CFQ_MON = Food Monitoring; CFQ_PROH = Food Prohibition.

Table S3.

Model comparison statistics for proposed, alternative, and parallel models.

Model	χ^2	df	CFI	TLI	RMSEA	SRMR	AIC	BIC
Full A	162.34	48	0.937	0.913	0.069	0.052	29738.144	29914.137
**Reduced A**	**163.60**	**50**	**0.938**	**0.918**	**0.067**	**0.051**	**29735.040**	**29903.012**
Full B	162.34	48	0.936	0.912	0.069	0.052	29738.144	29914.137
Reduced B	163.50	49	0.936	0.913	0.069	0.052	29737.297	29909.100
Full C	154.53	48	0.937	0.913	0.069	0.056	29738.144	29914.137
Reduced C	155.08	50	0.936	0.916	0.068	0.053	29735.132	29902.744
Parallel D	201.30	50	0.915	0.888	0.079	0.081	29773.105	29940.718
Parallel E	425.35	50	0.789	0.722	0.124	0.133	29997.149	30164.762

Note. All indices are based on robust (Satorra–Bentler) corrections. Smaller values of χ^2, AIC, and BIC and larger values of CFI and TLI reflect better fit. The proposed reduced Model A was retained for interpretation because it provided the best balance of *a-priori* parsimony and fit, while alternative recursive orderings yielded virtually identical global fit indices, and two parallel models yielded the poorest fits. Model A (full model with Dysregulated Maternal Eating as antecedent, Child Pro-Food Drive and Maternal Worry as sequential mediators in that order, and Maternal Control as the outcome); Model AR (Model A Reduced by removing nonsignificant paths from Dysregulated Maternal Eating to Maternal Worry and to Maternal Control); Model B (full, with the order of the mediators switched); Model BR (Model B reduced by removing nonsignificant paths from Dysregulated Maternal Eating to Maternal Control and from Maternal Worry to Child Pro-Food Drive); Model C (like Model A, but with Moder Worry as mediator of Pro-Food Drive); Model CR (Model C by removing nonsignificant paths from Dysregulated Maternal Eating to Maternal Control and from); Parallel D = with Dysregulated Maternal Eating as antecedent and Maternal Control as outcome; Parallel E = with Dysregulated Maternal Eating as antecedent and Child Pro-Food Drive as the outcome.

Table S4

Measurement Invariance of the Reduced Measurement Model Across Boys and Girls.

Model	χ^2	df	CFI	TLI	RMSEA	SRMR	ΔCFI	ΔRMSEA
Configural	220.85	104	0.937	0.920	0.067	0.059	—	—
Metric	233.11	112	0.935	0.923	0.065	0.064	−0.002	−0.002
Scalar	239.64	120	0.936	0.929	0.063	0.065		

Note. R-CFI = Robust Comparative Fit Index; R-TLI = Robust Tucker–Lewis Index; R-RMSEA = Robust Root Mean Square Error of Approximation; SRMR = Standardized Root Mean Square Residual; CI = confidence interval. *** $p < 0.001$. Model fit was evaluated using standard criteria: CFI and TLI ≥ 0.90 (preferably ≥ 0.95), RMSEA ≤ 0.08 (≤0.06 for close fit), and SRMR ≤ 0.08.
